# METACARPAL FRACTURES TREATMENT: COMPARASION BETWEEN KIRSCHNER WIRE AND INTRAMEDULLARY SCREW

**DOI:** 10.1590/1413-785220233103e266948

**Published:** 2023-09-08

**Authors:** Bruno Cesar Silva de Jesus, Clóvis Rodrigo Guimarães Braz Pereira da Silva, Rodrigo Domiciano Cardoso, Vitor Augusto Queiroz Mauad, Rafael Saleme Alves, Fernando Nogueira Zambone Pinto

**Affiliations:** 1 Faculdade de Medicina do ABC (FMABC), Orthopedics and Traumatology, Santo André, Sao Paulo, Brazil.; 2 Faculdade de Medicina do ABC (FMABC), Hand Surgery and Microsurgery, Santo André, Sao Paulo, Brazil.; 3 Faculdade de Medicina do ABC (FMABC), Santo André, Sao Paulo, Brazil.

**Keywords:** Bone Fracture, Metacarpus, Kirschner Wires, Fracture Fixation, Intramedullary, Trauma, Fratura, Metacarpo, Fios de Kirschner, Fixação Intramedular de Fraturas, Trauma

## Abstract

**Introduction:**

Metacarpal fractures are common and can be treated surgically using Kirschner wires (K-wires) or intramedullary fixation with compression screws (IMCS).

**Objectives:**

Analyze the postsurgical results from treating the metacarpal extra-articular fractures through the retrograde Kirschner wire technique, and compare it with the intramedullary compression screw fixation. Methods: Retrospective and quantitative studies were to analyze patients’ medical records, and a postsurgical evaluation questionnaire was given to the patients, who were divided into K-wire and IMCS.

**Results:**

The period of immobilization with a splint took six weeks for the K-wire group and four weeks for the IMCS group. The average time for consolidation took, respectively, fifty-seven days and forty-seven days. The first group could restart their activities twenty-two days after the other, and the average force value of the treated hand, when compared with its contralateral, was 93.9% and 95.4%, respectively. Between the operated hand and its contralateral, there was a difference of 16° in the total measures of the metacarpophalangeal and interphalangeal joint's range of movement among the K-wire group and 5° among the IMCS group.

**Conclusion:**

The patients who participated in this study showed excellent results after surgery, and both treatments were proven to be safe and reliable. ***Evidence level III; Retrospective comparative study*** .

## RESUMO

**Introdução:**

Fraturas dos metacarpos são frequentes e podem ser tratadas de forma cirúrgica com os fios de Kirschner (FK) e Fixação Intramedular com Parafuso de Compressão (FIPC).

**Objetivo:**

Analisar os resultados pós-operatórios do tratamento das fraturas extra-articulares dos metacarpos pela técnica retrógrada com fios de Kirschner e comparar com a fixação intramedular utilizando parafuso de compressão.

**Métodos:**

Estudo retrospectivo, quantitativo, com análise de prontuários, utilizando questionários de avaliação pós-operatória em dez pacientes divididos em dois grupos: FIPC e FK.

**Resultados:**

O período de imobilização com tala nos grupos FK e FIPC foram de seis e quatro semanas respectivamente, já o tempo médio para consolidação foi de 57 e 47 dias respectivamente. O grupo FK retornou as atividades laborais após os FIPC. O valor médio de força na mão acometida comparada a contralateral foi de 93,9% no grupo FK, e no FIPC de 95,4%. Medidas da soma de amplitude de movimento das articulações metacarpofalangeanas e interfalangeanas no grupo FK obtiveram diferença média entre as mãos operada e a contralateral de 16°, já na FIPC observou-se 5°.

**Conclusão:**

Os pacientes estudados apresentaram excelentes resultados pós-operatórios e ambos os tratamentos provam ser seguros e confiáveis. ***Nível de evidência III; Estudo retrospectivo comparativo*** .

## INTRODUCTION

Metacarpal fractures are very frequent and account for 36 to 42% of all hand injuries.^1 – 2^ Conservative treatment can be administered in cases where the fractures are stable. However, surgery is recommended when fractures show a rotational deviation of more than 5°, shortenings of more than 6mm, pseudo-clawing, or a variable angular deviation (depending on the injured metacarpus), because these deformities are related to significant biomechanical constraints on the efficiency of the flexor tendon and on the extensor mechanism of the fingers, and therefore can leave the patient with sequelae or other limitations in case these injures are not properly attended.^3 – 4^

Several surgical techniques are employed to heal these fractures, among which the Kirschner wires are the most popular implants not only for intra-articular fractures, but also for extra-articular ones. Some of the advantages of this technique are its low cost (when compared with other alternatives), and the fact that it is a percutaneous procedure. Besides, it also shows a lower rate of tendon adhesion.^5^ Nevertheless, it is still necessary to immobilize the affected area after surgery in order to protect it, but this prevents fracture from early rehabilitation.^6 – 7^

Regarding the intramedullary compression screws, it is agreed that they have proven to be a promising technique to treat diaphyseal fractures as well as stable fractures of the metacarpal neck without comminution.^8 – 9^ In addition to being a low-complexity method, they are also minimally invasive and, due to their steadiness, they do provide an early rehabilitation avoiding possible stiffness.^10 – 11^

Consequently, the objective of this study is to analyze the postsurgical results obtained from the treatment of the metacarpal extra-articular fractures through the retrograde Kirschner wire technique, and compare it with the intramedullary compression screw fixation.

## METHODS

This is a retrospective, comparative, descriptive and quantitative study, which offers an evaluation of medical records through the administration of clinical tests and surveys to assess the postsurgical condition of the patients who presented metacarpal fractures and were then treated surgically at the same medical center from 2019 to 2021. All the patients signed an Informed Consent Form, and the research was approved by the Local Research Ethics Committee (CAAE: 56459722.0.0000.0082).

The epidemiological data, which were descriptively analyzed, included the variables: age, gender, occupation, and the characteristics of both the fracture and the main limb. The time span for the radiological consolidation of the fracture was examined, as well as the time needed for patients to return to their habitual activities, and the time allocated for immobilization and possible postoperative complications. Postsurgical assessments were performed in order to examine function, mobility and strength flaws.

Ten patients who presented extra-articular metacarpal deviated fractures participated in this study and were divided in two groups, according to the surgical treatment to be administered: five of them joined the Kirschner wires (K-wire) group and the other five joined the intramedullary compression screw fixation (IMCS) group.

Patients who presented open metacarpal fractures, pathologic fractures, previous upper-limb injuries that showed sequelae, articular fractures or bilateral fractures, and patients who refused to be submitted to any of the procedures mentioned above were excluded from this research.

### Surgical Technique

In patients belonging to the IMCS group, a longitudinal incision of approximately 1cm was made in dorsal topography of the head of the fractured metacarpus. Another longitudinal incision was made in the extensor tendon, which provided a clear view of the metacarpal distal articulation. After that, a close reduction of the fracture was also made by longitudinal traction associated to the Jahss maneuver, followed by the introduction of the guide wire into the medullary canal, milling with a 2.7mm cannulated drill and the insertion of a 3.5mm medullary compression screw, placed 2mm below the articular surface. The patients were immobilized with a palmar splint for a few weeks, depending on their recovery process, and then sent to rehabilitation.

In patients belonging to the K-wire group, the retrograde introduction technique was employed. At first, a close reduction of the fracture was made by traction associated to the Jahss maneuver, followed by the medullary and crossed insertion of two Kirschner percutaneous wires measuring from 1 to 1.5mm. Due to the protocol on avoiding the risk of losing fracture reduction, patients of the K-wire group were immobilized with a splint for six weeks.

The patients were evaluated at least six months after the operation. The extension and flexion range of motion of the metacarpophalangeal joints, and of the proximal and distal interphalangeal joints (Total Active Motion – TAM) of all fingers was tested. Due to individual variation of finger extension and flexion, a comparison with the range of motion of the same contralateral finger, which was healthy, was made. Based on the criteria proposed in other studies,^12^ an excellent range of motion after surgery consists of less than 40° of the finger flexion's total loss, and less than 10° of the proximal interphalangeal joint's total loss (when compared with the healthy contralateral finger). A good range of motion presents a finger flexion's total loss varying from 40° to 80°, and a proximal interphalangeal joint's total loss between 10° and 30°. A poor range of motion includes a finger flexion's total loss higher than 80°, whereas the loss of the proximal interphalangeal joint is higher than 30° (when compared with the healthy contralateral finger).

The average handgrip strength of the injured limb and its contralateral was measured after three tests were carried out using a handgrip dynamometer (SAEHAN), then the strength was corrected by dominance.^13^ The rotational deformity was measured individually for each one of the injured fingers. In order to make an assessment of the upper limbs’ functionality after surgery, the following questionnaire was used: Disabilities of the Arm, Shoulder and Hand Questionnaire (DASH).^14^

### Statistical Treatment

All data were recorded using the software Microsoft Excel, and because there were not many samples, they were considered nonparametric. Therefore, they were described according to their proportion, median and interval – depending on the nature of the variables. Inferential tests were done through the χ^2^ – Mann-Whitney test – again, depending on the nature of the variables. These variables are represented in box plot graphs.

## RESULTS

For better understanding of the results, table 1 was prepared.

**Table 1:** Comparison between the groups.

	K-wire	IMCS
Age (interval)	30 years (15-47 years)	32 years (23-39 years)
Gender (Men:Women)	05:00	04:01
Trauma of the dominant hand	40%	80%
Smoker	40%	60%
Average duration of immobilization after surgery	6 weeks (±0)	4 weeks e 4 days (± 30 days)
Average time span for radiographic	57 days (± 16)	47 days (±7,6)
Average time span for patients to go back to work *	2 months e 22 days (± 13 days)	2 months (±36 days)
Results of limb functionality (0-100 points in DASH)	2 (±2,1)	2,5 (±2,8)
Rotational deviation	0	0
% of postsurgical handgrip strength (interval) **	93,9% (84,9-99,9%)	95,4% (87,9-110%)
Number of patients presenting loss in the range of motion (TAM) ***	2	1
Evaluation results of the range of motion (TAM) ****		
Normal hand	280 (±13,6)	286 (±10,2)
Postsurgical hand	264 (±15)	281 (±12,8)
% of patients who showed an excellent recovery after surgery	80% (4/5)	100% (5/5)

* One member of the K-wire group went back to work seven months later because of Social Security-related issues at the Brazilian Social Security Institute (INSS); One member of the IMCS group was unemployed when she got the injury.

** % of postsurgical handgrip strength when compared with the contralateral hand, having corrected its strength bearing in mind the limb dominance.

*** Patients belonging to the K-wire group showed losses of 30° and 48°; the only patient belonging to the IMCS group who showed a loss of 25° had a surgery-related complication (intra-articular screw fixation).

**** TAM – total range of motion (extension and flexion) of the metacarpophalangeal joints, and of the proximal and distal interphalangeal joints.

In the K-wire group, patients were, on average, 30 years old (ranging from 15 to 47 years of age), whereas patients in the IMCS group were 32 years, on average (ranging from 23 to 39 years of age) (p.0.69). The K-wire group consisted exclusively of men, but one woman participated in the IMCS group. The samples from both groups revealed four isolated fractures of the fifth metacarpus, and one isolated fracture of the fourth metacarpus. These fractures were present in the dominant hand in 40% of the patients of the K-wire group, and in 80% of the patients of the IMCS group (p 0.167).

The immobilization period with a splint was the same for all the patients in the K-wire group and lasted six weeks. On the other hand, the average period lasted 33.4 days for patients of the IMCS group (0-90 days). The average time span for the consolidation of the fractures in the first group lasted 57 days, but lasted 10 days less in the second group (p 0.643).

Seven months after the surgery, all the patients who participated in this research had already been back to their jobs, except for one patient who was unemployed. A K-wire patient who used to work in logistics took 210 days to get back to their activities, because their return was delayed due to Social Security issues at the Brazilian Social Security Institute (INSS). The fracture of this patient took 83 days to be consolidated. As sensitivity analysis, the assessment was carried out again excluding this specific case. As a result, the time needed for patients of the K-wire group to return to their activities was, on average, 22 days longer than the ICMS group (p 0.771).

The assessment of the upper limb's functionality was made through the DASH questionnaire, and its average result was 1.99 points (0-5.83) for the K-wire group, and 2,48 points (0-7.5) for the IMCS group (p 0.952). Regarding the percentage of handgrip strength of the operated hand when compared with its contralateral after the strength correction, the K-wire group showed 93,9% strength on the injured hand. In the other group, however, it was 95,4%, also when compared with its contralateral.

In patients of the K-wire group, the total measures of the range of movement of the metacarpophalangeal, and the proximal and distal interphalangeal joints (TAM) were 264° on the injured hand, an 280° on the contralateral hand, which suggests an excellent outcome in terms of postsurgical range of motion in 80% of the patients. In the IMCS group, the result of the total active motion was 281° on the injured hand, and 286° on the contralateral hand, which also suggests and excellent outcome in terms of postsurgical range of motion in 100% of the patients (p 0.444).

**Figure 1:**
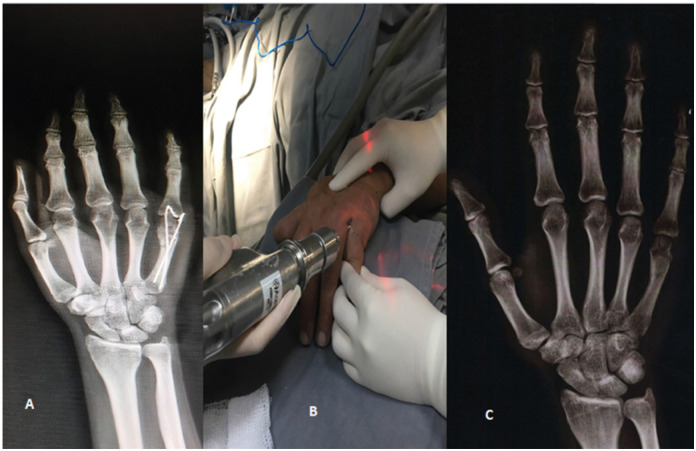
A) Anteroposterior radiograph in the immediate postoperative period of fixation of the 5° metacarpal diaphyseal fracture with FK. B) Retrograde intramedullary fixation technique with FK. C) Anteroposterior radiograph - Consolidation of the 5° metacarpal diaphyseal fracture.

**Figure 2:**
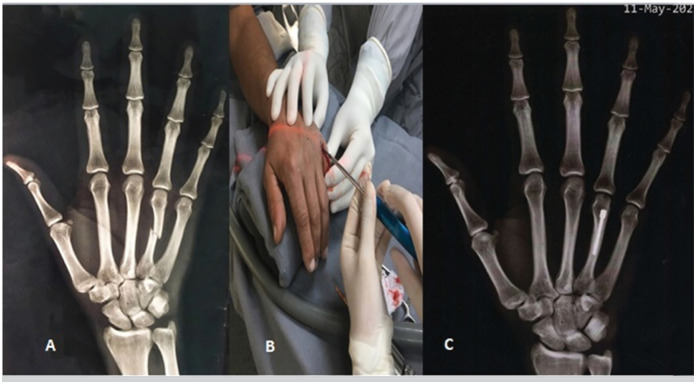
A) Anteroposterior radiograph – Diaphyseal fracture of the 4° metacarpal. B) IMCS surgical technique. C) Anteroposterior radiograph–Consolidation of the 4° metacarpal diaphyseal fracture.

Next, the FK and IMCS techniques will be represented, respectively, in figures 1 and 2 . The images represent the right hand in both cases of the figures.

## DISCUSSION

Most diaphyseal fractures and fractures of the metacarpal neck can be treated conservatively, as the angle between them and the dorsal apex can be functionally compensated by a 20°-30° motion of the ring and little fingers carpometacarpal joints. There is no consensus on the acceptable degree of an angular deviation for metacarpal neck fractures of these two fingers.

Previous researches, however, have reached satisfactory clinical results for conservative treatments showing volar angulations between 30 to 70 degrees.^15^ Nevertheless, research done on corpses have suggested that a flaw on the metacarpal neck whose dorsal apex is higher than 30° cause a decrease in length and function of the intrinsic muscles, and reduce the efficiency of the flexor system throughout the metacarpophalangeal joints mobility.^16^ As for the middle and index fingers, a reduction in and stabilization of diaphyseal fractures, and of fractures of the metacarpal necks that present abnormalities higher than 10°-15° in their sagittal plane are essential, since their carpometacarpal joints are rigid. Pseudo-clawing and rotational deformities are also suitable for surgical treatment in order to reduce and stabilize the fracture.

Several surgical techniques have been applied to treat diaphyseal fractures, metacarpal subcapital fractures and fractures of the metacarpal neck that present significant deviations. It has not been decided on which surgical technique is the best, though nowadays one should take the characteristics of the fracture and the surgeon's preference into consideration before opting for a particular treatment.

Concerning the percutaneous Kirschner-wire technique, it avoids tissue lesion, but demands postsurgical immobilization for a period that varies from four to six weeks, so that any loss in reduction can be prevented. In this study, no complications involving K-wire patients were brought to our attention. Nonetheless, two in-depth studies have shown predominance of 16% in postoperative complications after treatments that resorted to Kirschner wires.^17^ Some of these complications included: osteomyelitis, rupture of the extensor tendon, neurological lesion and pin-tract infection. In addition to that, a randomized clinical trial did not reveal any differences in clinical results after comparing the conservative treatment of the metacarpal neck fracture on the fifth metacarpal bone with the percutaneous fixation with Kirschner wires.^18^ On the other hand, another randomized clinical trial concluded that there are no differences between the treatment of boxer's fractures with either the Kirschner wires or the intramedullary screw fixation, as excellent results were achieved and there were just few complications in both groups.^19^

This research, however, manifests a surgical complication in one of the IMCS group patients, who complained about the pain and who had a significant decrease in the range of motion two months after the surgery that treated a diaphyseal fracture in the fifth metacarpal bone on their right hand. After six months, this patient was submitted to another surgery to remove the screw, which was found in intra-articular position. By doing so, there was a considerable improvement in the range of motion, and an ease of the pain. In spite of this, the present research also demonstrates that, after more than six months, the patients who underwent either the treatment with Kirschner wires or with intramedullary screw fixation showed good results in terms of range of motion, strength, pain relief and functionality (according to the DASH questionnaire). Besides, all fractures healed properly, and patients could go back to their daily activities and to their jobs. No significant differences relevant to statistics were identified among the patients of the two groups.

It is believed that intramedullary compression screws are better suited for cases where there is no comminution, for the introduction of the screws into comminuted fractures can lead to bone shortening. Screw fixation usually present good results in patients who have demanding jobs and need to return to their activities shortly. However, this synthesis can be a support,^20^ but will not provide the same level of stability that the fixation of locking plates does. For this reason, it is important to be wary of the postsurgical rehabilitation protocol, in order to avoid rotational deviation and fracture reduction loss. In addition to that, studies on long-term prospective monitoring to assess metacarpophalangeal joints sequelae are inexistent, which makes it hard to establish how safe this technique really is. In order for one to recommend this technique to treat metacarpal fractures, then head, neck and diaphyseal fractures that are not comminuted should be its main indicators.

This study also has some shortcomings, such as the difficulty to follow the postsurgical rehabilitation protocol due to patient's unavailability to attend physiotherapy sessions. Furthermore, the samples used in this research contained only fractures of the ring and little fingers, which interferes with the projection of other results related to extra-articular fractures of the other metacarpi; thus, the amount of samples is limited.

## CONCLUSION

In conclusion, this study demonstrates that patients who underwent osteosynthesis to treat a metacarpal extra-articular fracture with the retrograde Kirschner wire technique or intramedullary compression screws fixation showed great postsurgical results in their range of motion and strength, and all of them could return to their usual activities and jobs. Consequently, taking the positive outcomes into consideration, new studies on this matter are strongly suggested, specially to assess these patients in the long term.
